# Efficacy of Edible and Leisure Reinforcers with Domestic Dogs

**DOI:** 10.3390/ani13193073

**Published:** 2023-09-30

**Authors:** Xenabeth A. Lazaro, John M. Winter, Jonathan K. Fernand, David J. Cox, Nicole R. Dorey

**Affiliations:** 1 Department of Psychology, University of Florida, Gainesville, FL 32611, USA; 2 Florida Institute of Technology, School of Behavior Analysis, Melbourne, FL 32901, USA; 3 Institute for Applied Behavioral Science, Endicott College, Beverly, MA 01915, USA; 4 RethinkFirst, Department of Data Science and Analytics, New York, NY 10001, USA

**Keywords:** reinforcer efficacy, edible reinforcer, leisure reinforcer, paired-stimulus preference assessment, progressive ratio, animal welfare, domestic dog, *Canis familiaris*

## Abstract

**Simple Summary:**

In this study, researchers aimed to compare the effectiveness of food and leisure stimuli as reinforcers for domestic dogs. While preference assessments have been conducted for various species, including humans and animals like cockroaches and wolves, to our knowledge, no study has specifically examined the preference between food and leisure stimuli in dogs. This study found that, overall, domestic dogs showed a preference for food over leisure items. Additionally, food was found to be a more effective reinforcer for dog behavior compared to leisure items. These findings have important implications for dog owners and trainers, suggesting that using food as a reinforcer may yield better results in training dogs.

**Abstract:**

Preference assessments are often used to identify stimuli that function as potential reinforcers for training or intervention purposes. Specifically, various preference assessment formats have been used to identify preferred stimuli for humans, cockroaches, cotton-top tamarins, tortoises, and wolves, to name a few. However, to date, no study has evaluated the differential efficacy between food and leisure stimuli within domestic dogs. The current study aimed to compare the reinforcing value and efficacy between food and leisure stimuli for domestic dogs by comparing rates of behavior when receiving access to either their top-preferred food or leisure items. Overall results suggest (1) domestic dogs prefer food over leisure items, and (2) food is more likely to function as a reinforcer than leisure items for domestic dog’s behavior. These results suggest that dog owners and trainers should consider using food reinforcers over leisure items as reinforcers when attempting to train dogs.

## 1. Introduction

Preference assessments have been established in behavior analysis with humans to identify potential reinforcers for several decades [1]. With animals, the applications can range from informing environmental conditions [2,3,4,5,6] and food preference [7,8,9,10,11] to further understanding of animal–human social interactions [12,13,14,15] and identifying toy or enrichment devices [16,17]. In domestic dogs, preference assessments have been used for food taste testing, although methods vary for dog food palatability tests (see [11] for a thorough review), comparing preference between different types of toys [18,19], and comparing preference between different reinforcers, such as food and human social interaction [14].

A paired-stimulus preference assessment (PSPA) allows comparisons of preference between stimuli by putting a series of paired stimuli in front of the participant and allowing access only to the stimulus the participant selects [20]. The PSPA method can be used as an effective way to compare a list of items in pairs with each other and has been shown to produce consistent results for identifying a hierarchy of effective reinforcers for humans [20,21,22,23,24], cockroaches [25], cotton-top tamarins [26], Galapagos tortoises [27], giant pandas and African elephants [28], domestic dogs [29,30], and wolves [13].

Although these tests might be useful for identifying preferences in various organisms, little research has been conducted on domestic dogs using PSPAs to identify possible reinforcers. Vicars [30] conducted a PSPA with eight dogs using six foods. The preferences of each dog were identified, and dogs were then given a task to investigate reinforcement efficacy. Progressive-ratio reinforcer (PR) assessments were also conducted to further study reinforcer efficacy. During this single-operant assessment, dogs were given one of the experimenter’s fists to touch with their muzzle to obtain either the highest or lowest preferred food across separate sessions (which were determined from the PSPA). The number of times they needed to touch the fist before receiving the food increased with every trial. Vicars [30] found that preference for an item predicted its reinforcer efficacy for dogs, illustrating that stimuli ranked higher in the PSPA might be more effective reinforcers than those ranked lower in the PSPA. 

Similarly, Cameron [29] applied the PSPA to eight dogs to determine if the outcome of the assessment could predict reinforcer efficacy. In this investigation, a dog’s preference was tested across six flavors of raw food and a portion of staple food (a specific raw food that was fed to the dogs by their owners for three days before testing) to identify a rank order of preference for the foods. After the PSPA was completed, the researchers ran a reinforcer assessment. In this study, the reinforcer assessment required dogs to walk down a 5-m runway to obtain their staple, most- or least-preferred foods. The investigators found that the PSPA did seem to give the correct rank order of preference and found that staple foods were not highly valued. Furthermore, the researchers found a significant difference in the speed at which the dogs ran the runway for highly preferred food compared to the staple of less preferred food, indicating that additional dimensions of behavior other than selection responses may be predictive of differential preference between stimuli. 

Although prior research supports the use of PSPAs to inform food preferences in dogs, no research has been conducted to assess leisure preferences. In addition, to date, no study has evaluated the differential efficacy between edible and leisure stimuli with domestic dogs. The purpose of this study was to compare the reinforcing value and efficacy of food and leisure items for domestic dogs by comparing rates of behavior when receiving access to either their top-preferred food or leisure item, using PSPAs to measure preference and a PR schedule to further assess reinforcer efficacy.

## 2. Methods

### 2.1. Subjects and Setting

A total of 10 pet dogs *(Canis lupus familiaris)* were recruited and tested for the study (see Table 1 for further information). The dogs were recruited via social media advertisements and word-of-mouth. Experimental sessions were conducted in an open area that was familiar to the dog and distraction-free. All sessions were videotaped with a Sony DCR-SX44 4GB Handycam Camcorder.

### 2.2. General Experimental Procedure and Materials

The owners were asked to refrain from feeding their dog(s) for at least 4 h prior to the experiment [30,31]. Six food items and six leisure items were chosen by researchers to be likely preferred food and leisure items for most dogs. The six different food items were hotdog, carrot, cheese, kibble, hard dog treat, and soft dog treat. Each piece of food was approximately a 1 cm by 1 cm cube. The six different leisure items were a ball, tug toy, squeak toy, bone, stuffed animal, and empty plastic water bottle. 

### 2.3. Preference Assessment Procedure

Subjects were given an exposure trial for each food and leisure item separately to allow each subject to sample and become familiar with the items before the preference assessments were conducted. For each exposure trial, the subjects were required to consume the food and interact (approximately 10 s) with each leisure item. During the PSPAs, subjects had 1 min to make a selection. A choice for food was defined as swallowing one food item. A choice for a leisure item was defined as the subject touching its muzzle to a leisure item. Each food and leisure item was counterbalanced across 15 trials, and the order of separate edible and leisure preference assessments was counterbalanced across subjects. Subjects with side biases that could not be corrected were precluded from the study.

During the preference assessment, the total number of times an item was selected was calculated, and the item with the largest percent selected was considered the highest preferred among its group of six items (i.e., edible and leisure). If two items within the same PSPA tied for highest preferred, those two items were pitted against each other, and two more preference assessment trials were conducted with those two items, swapping places to counterbalance in those two trials. Again, the highest preferred food from the edible PSPA and the highest preferred toy from the leisure PSPA were used as the highest preferred items within the experimental trials. 

After the completion of both preference assessments (i.e., edible and leisure), each subject was trained using the third highest preferred food to touch their muzzle to the experimenter’s fist. When the subject could reliably walk 1 m to touch their muzzle to the experimenter’s fist, training was considered complete, and the experimental phases were conducted.

#### 2.3.1. Food Preference Assessment

**Figure 1:**
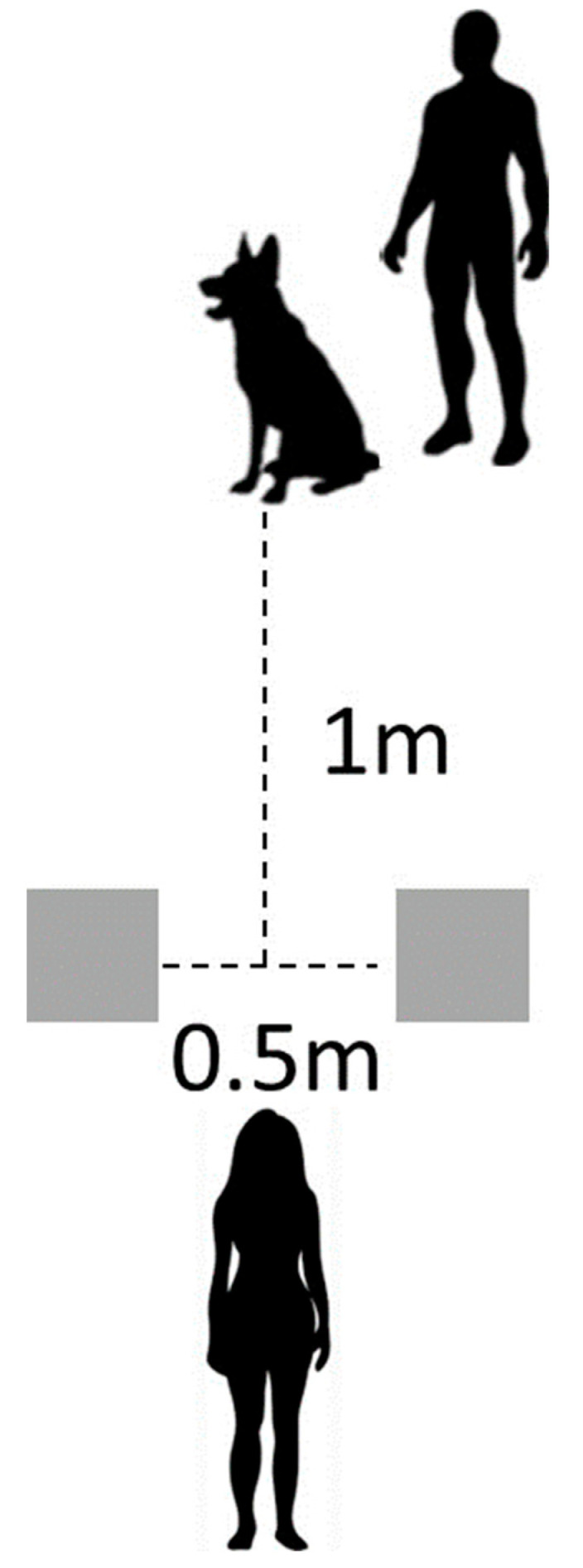
Experimenter places baited cardboard squares 0.5 m from each other and 1 m away from subject and handler before giving the cue “subject’s name, look at me, ok.”.

During the food preference assessment, white gloves were worn as a discriminative stimulus to indicate food. Preference assessments began with the subject 1 m away from the experimenter. In the food preference assessment, food was placed simultaneously on two cardboard squares (5 in × 5 in). The two squares were 0.5 m apart from each other, equidistant to the subject (see Figure 1). The preference assessment began with the experimenter saying the subject’s name and “look at me” to get its attention. When the experimenter said “ok”, the subject was released to approach and consume one of the food pieces. As soon as the subject consumed one of the food items, the remaining food item was removed, and the subject was reset to begin the next trial. If the subject did not choose either food item, it was reset once, and the same trial was repeated. If the subject did not choose for a second time, the trial was marked as “No Choice”, and the next trial was conducted. 

#### 2.3.2. Leisure Item Preference Assessment

In the leisure item preference assessment, black gloves were worn as a discriminative stimulus to indicate a leisure item trial. The experimenter stood the same distance from the subject as in the food preference assessment. To present the leisure items, the experimenter squatted and held out both hands, shoulder width apart from each other, with a different leisure item in each hand, shaking both leisure items slightly. The preference assessment began with the experimenter saying the subject’s name and “look at me” to get its attention. When the experimenter said “ok”, the subject was released to approach and choose one of the leisure items. When a leisure item was selected, the experimenter allowed the subject to play with the leisure item as typically intended (i.e., the tennis ball was thrown, or the rope was used in a game of tug-of-war) and removed the other leisure item. The subject was allowed to play with the selected leisure item for approximately 10 s without human interaction before being reset to the starting position for the next trial. “No Choice” was marked as previously described in the food preference assessment.

### 2.4. ABCABC Phase

#### 2.4.1. Design

In this phase, three different conditions were conducted: contingent edible reinforcer, in which the dog received its most preferred food as a reward; contingent leisure reinforcer, in which the dog received its most preferred leisure item as a reward; and extinction, in which the dog was shown an empty hand with no reward. The design was an ABCABC reversal design to compare the reinforcement effects between the edible and leisure items. Conditions A and B were contingent edible reinforcer and contingent leisure reinforcer counterbalanced across subjects (see Table 1), but Condition C was always extinction. Conditions A and B were counterbalanced so that half of the subjects had Condition A as a contingent edible reinforcer and Condition B as a contingent leisure reinforcer, and the other half of the subjects had Condition A as a leisure reinforcer and Condition B as a contingent edible reinforcer. Each subject was asked to perform the target behavior for each trial to receive the reward. A response was defined as the subject touching its muzzle to the experimenter’s outstretched, closed fist. 

In each of the three conditions, there were three sessions containing a maximum of fifteen trials. Each was conducted in a similar manner as Experiment 1 in Feuerbacher and Wynne [14]. When a subject reached the maximum of 15 trials in a session or reached termination criteria, the next session began. Termination behavior consists of 1 min of non-responding, the subject walking 2 m away from the experimenter for 5 s, or the subject placing its head on the floor. During each trial, if a subject did not respond for 30 s, it was re-oriented to be 1 m from the experimenter, and the experimenter re-presented the gloved fist by lifting it up, saying the subject’s name, “look at me, ok”, and placing the fist back down at level with the subject’s muzzle. If the subject still did not respond for another 30 s, the termination criterion was met (i.e., a full 60 s of non-responding), and the next session began.

During each condition, a different color glove was worn. When the condition offered food as a reward, white gloves were worn; when the condition offered a leisure item as a reward, black gloves were worn; and when the condition offered no reward (i.e., operant extinction), blue gloves were worn.

#### 2.4.2. Contingent Edible Reinforcer

**Figure 2:**
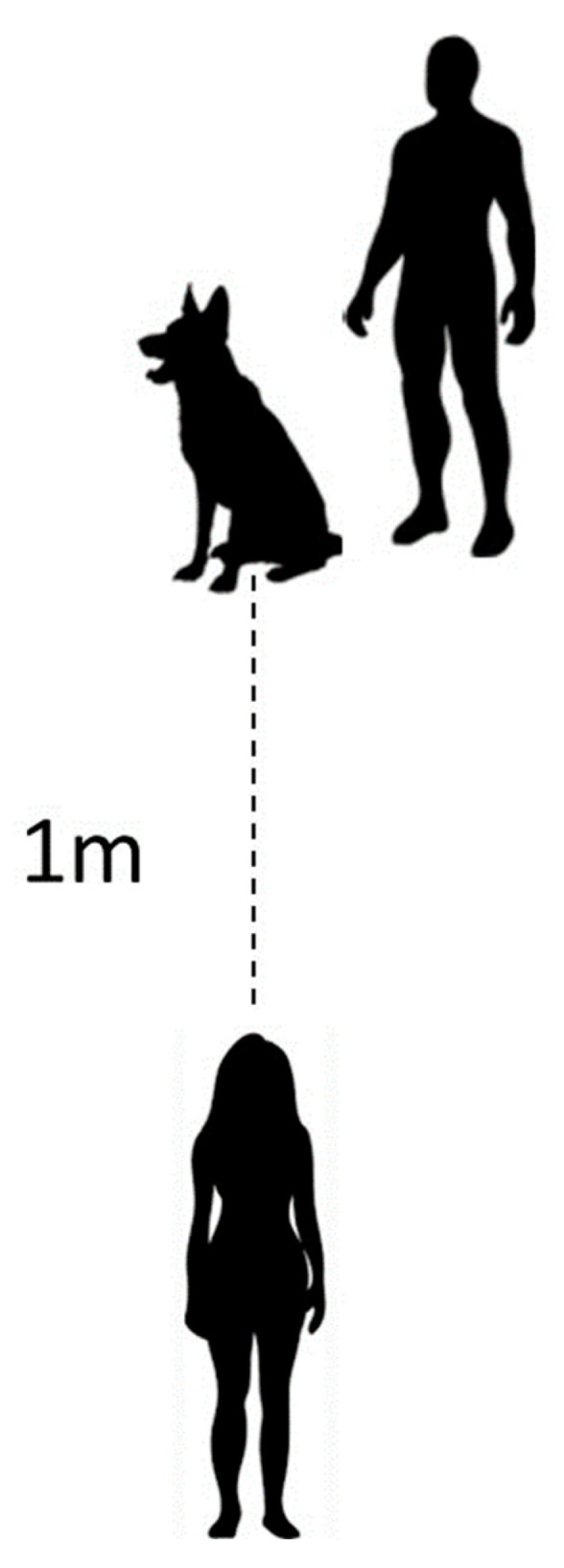
Experimenter stands 1 m away from subject and handler. Subject must walk 1 m and perform behavioral tasks to receive reinforcement (i.e., preferred food, preferred leisure item, or extinction).

Similar to the preference assessment procedure, each trial began with the subject 1 m away from the experimenter. However, in this phase, the experimenter would stretch out their closed fist at level with the subject’s muzzle, with the reward held in the other hand hidden behind the experimenter’s back (see Figure 2). The experimenter would call the subject’s name, say “look at me”, and then say “ok” to signal that the subject was free to touch their hand. If the subject performed the target behavior (i.e., approached the hand and touched it with their muzzle), the reward was given to them. The food was delivered by bringing it from behind the experimenter’s back directly in front of the subject’s muzzle on an open palm. After consumption of the food, the next trial was set up and begun.

#### 2.4.3. Contingent Leisure Reinforcer

These sessions were conducted exactly in the same way as the contingent edible reinforcer sessions, with the exception that a leisure item was delivered contingent upon the subject performing the target behavior. After 10 s of play with the leisure item, the next trial was conducted.

#### 2.4.4. Extinction

Extinction sessions were conducted in the same manner, with the exception that extinction was in place for the target behavior. That is, the experimenter brought their other hand from behind their back and opened their palm to show an empty palm. After a few seconds of showing the subject the empty palm, the next trial was set up, and the session continued.

### 2.5. Progressive-Ratio (PR) Phase

#### 2.5.1. Design

In the last phase, separate PR trials were conducted with the high-preferred food and leisure items as a further comparison of the effects of reinforcement under leaner schedules (similar to [30] design with low- and high-preferred foods). Each condition had three sessions: three food sessions and three leisure item sessions. A programmed extinction condition was not conducted for this phase. In each condition, all three sessions were completed before moving on to the next condition. The order of these conditions was counterbalanced across subjects, correlating with the ABCABC phase for that individual. If Condition A was a contingent edible reinforcer for the subject in the ABCABC phase, trials for the PR schedule began with the high-preferred food first, followed by the PR schedule with the high-preferred leisure item. If Condition A was a contingent leisure reinforcer for the subject during the ABCABC phase, that same individual was given the leisure item trials first in the PR schedule. Sessions were terminated when the subject met the termination criteria listed in the ABCABC phase section above.

Just as in the ABCABC phase, a white glove was worn when food was to be delivered, and a black glove was worn when delivering the leisure item.

#### 2.5.2. Procedure

PR trials began the exact same way the ABCABC trials did (see Figure 2). The subject was positioned 1 m away from the experimenter, and the experimenter would extend their closed fist and say the subject’s name, “look at me, ok”. For the first trial, if the subject performed the muzzle touch once, it received the reward (an FR1 schedule). Where the PR phase differed from the ABCABC phase was that reinforcement was rapidly thinned on subsequent trials when the subject emitted further responses (e.g., trial 1 = 1 muzzle touch, trial 2 = 2 muzzle touches, trial 3 = 3 muzzle touches, etc.), but the reward remained the same. To create clear and separate touches, after the subject initially touched the fist, its muzzle had to move away from the experimenter’s hand at least 2.5 cm before another touch could be counted. 

For the termination criterion of 1 min of non-responding, the minute was reset every time the subject made a new touch. For example, if the subject had gone 40 s without responding and then touched its muzzle to the experimenter’s fist, they would get another 60 s to touch the fist again before the termination criterion would be met. The experimenter would still prompt the subject after every 30 s of non-responding by re-orienting them, removing the gloved hand, then offering the gloved hand again while saying the subject’s name, “look at me, ok”.

## 3. Results

**Figure 3:**
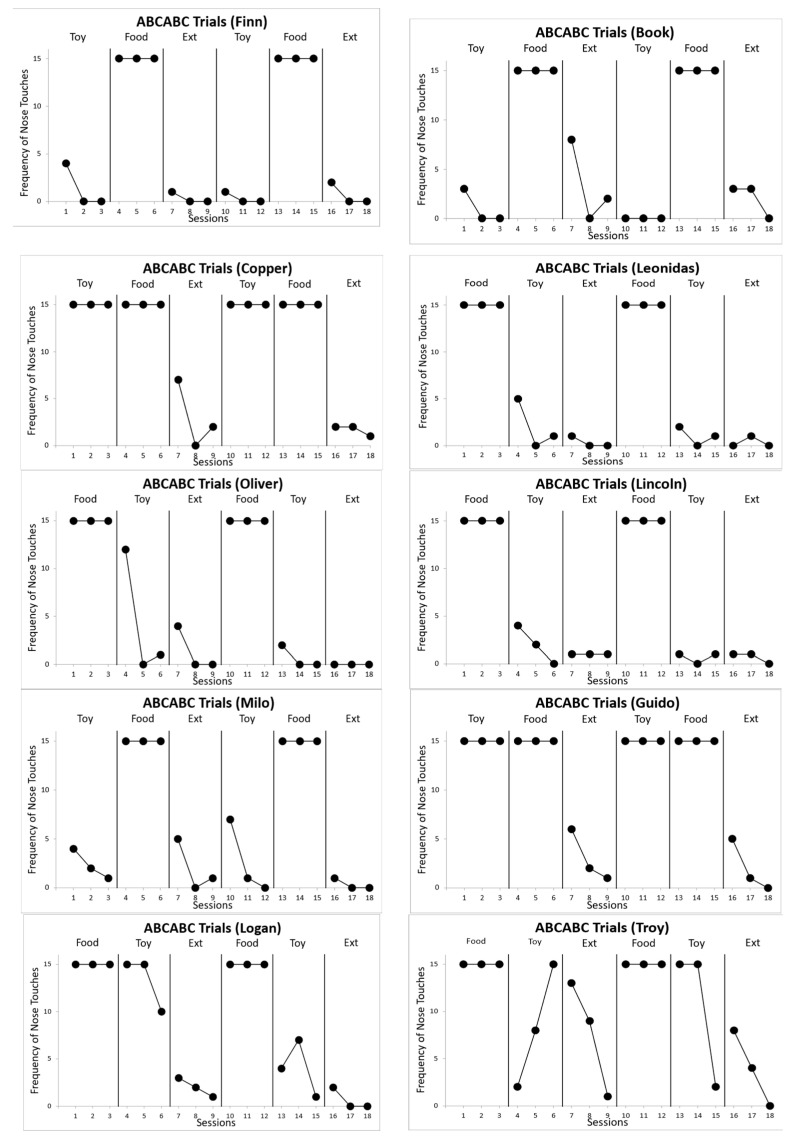
Frequency of nose touches across sessions for each subject in the ABCABC reversal phase.

In the ABCABC phase during the edible reinforcer condition, all subjects reached 15 trials. Whereas during the contingent leisure reinforcer and extinction conditions, subjects rarely reached 15 trials. Only two subjects, Copper and Guido, reached fifteen trials in both the contingent edible and leisure reinforcer conditions (Figure 3). 

**Figure 4:**
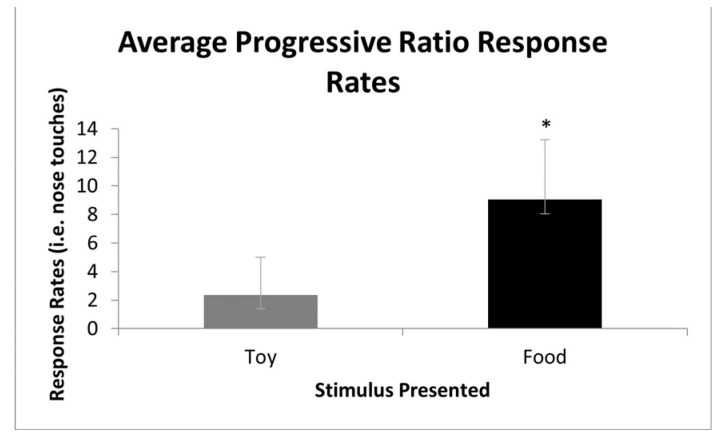
Overall average response rates for the contingent leisure and edible reinforcer stimuli during progressive-ratio sessions across subjects. * *p* ≤ 0.001.

A statistically significant difference in response rates was found in the PR sessions between the two reinforcer conditions ( paired *t*-tests, t(30) = −6.32, *p* ≤ 0.001; see Figure 4). This demonstrates a significant preference for the efficacy of edible reinforcers over that of leisure reinforcers across subjects.

**Figure 5:**
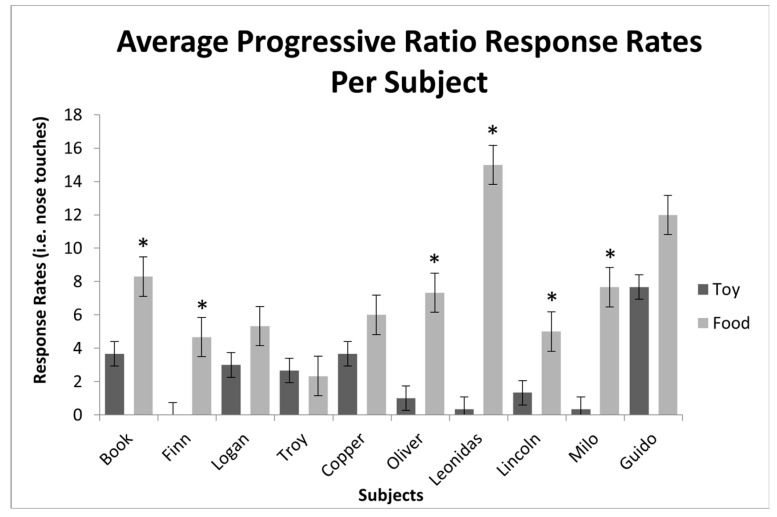
Average of the three sessions for the contingent leisure and edible reinforcer stimuli per subject across all progressive-ratio sessions. * *p* ≤ 0.001.

Out of the ten subjects, nine (90%) responded more to the edible reinforcer condition than the leisure reinforcer condition during the PR trials (Figure 5). The exception to this was Troy, who had a higher average response rate for the leisure condition than for the edible condition; however, the difference was not found to be significant. Additionally, seven of the ten subjects (70%) had statistically significant differences between response rates for each condition, with the exception of Logan, Troy, and Copper, whose differences were not significant.

## 4. Discussion

This study is the first, to our knowledge, to compare the efficacy of edible and leisure reinforcers in domestic dogs. Although Feuerbacher and Wynne [14] had similar findings when comparing the preference for food with that of human interaction. In our study, results of the reinforcement efficacy (i.e., ABCABC) trials indicated that dogs preferred food as a reinforcer and were more motivated to work for food over a leisure item. Even subjects that equally enjoyed food and leisure items during the reinforcement efficacy trials, when required to work for the reward on a leaner schedule, demonstrated that they were more motivated by their favorite food than their favorite leisure item. Although edible reinforcers were likely to function as more potent reinforcers over leisure items, these results still confirm that leisure items still functioned as reinforcers for many dogs as responding for leisure items was still higher than when receiving no consequence (i.e., extinction); however, dogs completed more trials under the contingent edible condition as well as the PR requirements when edible reinforcers were used relative to the leisure item condition. It should be noted that we evaluated the effects of edible and leisure reinforcers in single-operant arrangements and were never compared in a concurrent arrangement. Prior research has indicated that less-preferred stimuli will likely not be selected when higher potent reinforcers are available concurrently; thus, when edible and leisure items are used in the same assessment, it is likely the participant will select the edible reinforcer [32,33]. Although this was not tested in the current study, this may be relevant in training contexts in which both edible and leisure items are available concurrently. 

These data could provide useful information for behavior modification or cognitive testing. Understanding the preferred reward might not be enough to motivate a subject. Using the PR schedule could inform further applied uses of these rewards in training situations. PR schedules are a viable method of testing the efficacy of reinforcers due to the subject’s task to perform a behavior on an exponential progression in order to receive a reward. By making the subject respond more repeatedly for a reward, PRs are capable of shining light on more information about differences in the efficacy of stimuli that would otherwise go unnoticed with other methods, such as PSPAs [34,35,36]. Cameron [34] compared food reward response rates in wild brushtail possums between PRs, fixed ratios (FR), and progressive fixed ratios (PFR). Their results suggested that average response rates were higher for the PFR than the PR schedules; however, all the conditions were still effective in testing food response rates. Of the three schedules, PR was suggested to be more potent and time-efficient when assessing response rates. Additionally, Vicars [30] investigated the effectiveness of high- versus low-preferred foods as reinforcers in dogs by using a PR schedule. This paradigm showed that when tasked to perform muzzle touches for a high- or low-preferred food, response rates increased for highly preferred foods. These examples indicate that PR schedules provide useful data on operant behavior, further evaluating the effects of reinforcers on performance. Adopting the leaner schedule of the PR paradigm into reinforcer efficacy testing can aid in identifying reinforcers and validating preferences identified by preference assessments solely based on the response rates of subjects. Furthermore, assessing breaking points within a PR schedule may inform how lean the schedule can be within training contexts to still maintain responding in animals. That is, an edible reinforcer may only need to be delivered every so often to maintain performance, whereas a leisure item may need to be delivered more frequently to maintain the same behavior and to the same quality. 

These findings are also important in understanding what individual canines are motivated to work for as this is a prerequisite to effectively shape behavior. Many dog trainers advocate toys in their training over food rewards [37,38]. Martuch [37] stated that leisure items can provide training advantages that food cannot by turning training into a fun activity, therefore creating a stronger drive, increasing confidence, and reducing the stress that can be caused by trying to learn a behavior. Martuch [37] also noted several potential disadvantages to using food as reinforcers, such as the dog getting too focused on the food, the dog getting bored with the same flavor, and having to cut meals from the dog to prevent it from gaining excess weight. Although these claims are echoed by other professional dog trainers, none of these claims have been backed by research. 

This study focused on comparing the reinforcing value and efficacy between food and leisure stimuli for domestic dogs by comparing rates of behavior. Further research is needed to investigate some of the specific claims touted by dog trainers. For example, Ryan [39] stated that the label of leisure item or food as the best reward depends on the training. For active commands like “come”, a leisure item could be useful to energize the dog and make the trainer seem interesting, but for a situation with more stationary commands like “down”, Ryan argues that food may be better. In addition, future research should investigate whether dogs that have a history of working for leisure items, like search and rescue dogs, will increase their rates of behavior for a preferred leisure item over a preferred food reward. 

## 5. Conclusions

This study aimed to compare the reinforcing value and efficacy between food and leisure stimuli for domestic dogs by comparing rates of behavior when receiving access to either their top-preferred food or leisure items. The results indicate that, overall, dogs exhibit a stronger preference for and food is more likely to function as a reinforcer than leisure items for domestic dog’s behavior. Even when dogs had equal preference for both types of stimuli in preference assessments, they showed a higher response rate when working for their preferred food during progressive-ratio trials.

These findings have practical implications for dog training. Understanding a dog’s preferred reward can be essential for shaping behavior effectively. Further research is warranted to explore how the choice between food and leisure items as rewards may depend on the specific training goals, the individual dog and behaviors being targeted. Additionally, investigating the preferences and reinforcement efficacy of working dogs with a history of responding to leisure items could shed light on their unique motivations and needs.

## Figures and Tables

**Table 1:** Subject information.

Subject	Sex	Breed	Age	First Condition	Preferred Food	Preferred Leisure Item
Book	♂ M	Mix	11 mo	Leisure	Hotdog	Water Bottle
Copper	♀ F	Mix	2 yrs	Leisure	Soft Treat	Stuffed Animal
Finn	♂ M	Mix	2 yrs	Leisure	Hotdog	Stuffed Animal
Guido	♂ M	German Shepherd	2 yrs	Leisure	Hard Treat	Ball
Leonidas	♂ M	Labrador	2 yrs	Edible	Hard Treat	Plastic Bone
Lincoln	♂ M	Mix	1 yr	Edible	Hard Treat	Ball
Logan	♀ F	Mix	3 yrs	Edible	Hard Treat	Stuffed Animal
Milo	♂ M	Mix	NA	Leisure	Soft Treat	Stuffed Animal
Oliver	♂ M	German Shorthaired Pointer	1 yr	Edible	Hard Treat	Ball
Troy	♂ M	German Shepherd	8 yrs	Edible	Carrot	Ball

## Data Availability

The data presented in this study are available upon request form the corresponding author.
